# A unique case of hemolytic‐uremic syndrome secondary to enteropathogenic *E Coli*

**DOI:** 10.1002/ccr3.3221

**Published:** 2020-08-14

**Authors:** Blessie Elizabeth Nelson, Angelina Hong, Fatima Iqbal, Bagi Jana

**Affiliations:** ^1^ Department of Hematology & Oncology University of Texas Medical Branch Galveston Texas USA; ^2^ School of Medicine University of Texas Medical Branch Galveston Texas USA; ^3^ Department of Pathology University of Texas Medical Branch Galveston Texas USA; ^4^ Department of Hematology & Oncology MD Anderson Cancer Center Houston Texas USA

**Keywords:** anemia, dialysis, enteropathogenic *E Coli*, hemolysis, Hemolytic‐uremic syndrome, thrombocytopenia

## Abstract

Causative factors of HUS due to infection are not limited to classic EHEC and Shigella infection. Understanding the effects of EPEC‐related HUS and its complications is imperative for early diagnosis and treatment to mitigate long‐term sequelae.

## Graphical abstract

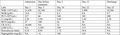

## 1 BACKGROUND

Typical form of hemolytic‐uremic syndrome is caused most commonly by enterohemorrhagic *E Coli* strain O157:H71. Here, we report a unique case of HUS secondary to enteropathogenic *E Coli* infection, with a discussion on the diagnosis of HUS and how it is distinguished from other thrombotic microangiopathies (TMAs).

Hemolytic‐uremic syndrome (HUS) is a potentially life‐threatening hematologic disorder that presents with the triad of microangiopathic anemia (MAHA), thrombocytopenia, and acute renal failure secondary to vascular damage. The typical form of HUS (tHUS) is caused by an *E Coli* or *Shigella* infection, most commonly enterohemorrhagic *E Coli* (EHEC) strain O157:H7.^1^ Atypical HUS (aHUS) is caused by a genetic mutation causing abnormal complement activation. Enteropathogenic *E Coli* (EPEC) is a well‐known cause of diarrhea in pediatric patients who live in developing countries, but is a rare cause of adult diarrhea, and has not been documented as a cause of HUS.^2^ Here, we report a unique case of HUS secondary to EPEC infection, with a discussion on the diagnosis of HUS and how it is distinguished from other thrombotic microangiopathies (TMA).

## 2 CASE REPORT

**FIGURE 1:**
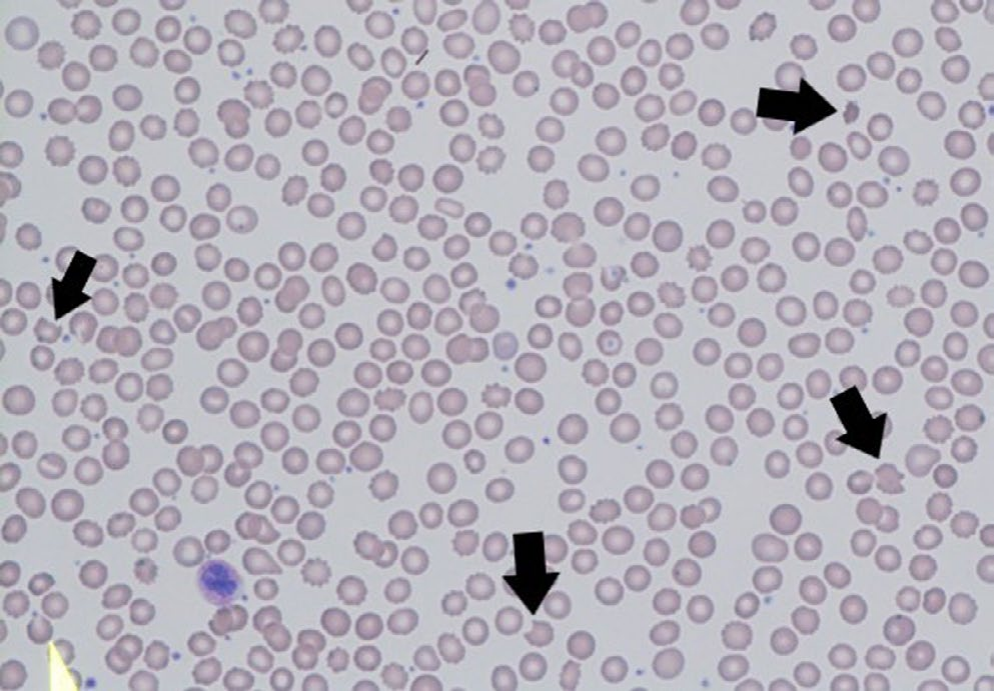
Blood smear depicting MAHA. Prior to treatment, the patient's blood smear showed approximately 7‐10 schistocytes (arrows) per high‐power field at 100× magnification. Following treatment, the patient's blood smear showed fewer schistocytes, about 0‐2 per high‐power field

A 64‐year‐old woman with a history of early‐stage, hormone‐positive breast cancer diagnosed in January 2019 presented with weakness, fatigue, nausea, vomiting, and watery, nonbloody diarrhea that started within 48 hours after eating restaurant food. In the emergency department, maximum temperature was 37.4°C (99.3°F), heart rate was 156, and blood pressure was 95/63. She was alert and oriented to person, place, time, and situation. Her white blood cell count (WBC) was 21 430/uL, hemoglobin (HGB) 14.8 g/dL, platelet count (PLT) 227 000/uL, potassium 3.5 mEq/L, lactate 8.68 mg/dL, creatinine 2.2 mg/dL, BUN 37 mg/dL, and creatine kinase (CK) 10 846 U/L. She was diagnosed with hypovolemic shock and acute kidney injury secondary to rhabdomyolysis. She was given aggressive IV hydration with empiric antibiotics and was sent to the ICU for higher level of care. Over the next 5 days, she developed severe thrombocytopenia and anemia, reaching a nadir PLT count of 50 (10*3/µL) and HGB of 5.0 (g/dL). She was anuric and transitioned from continuous renal replacement therapy to conventional hemodialysis. Complement levels were low during this acute stress state. Blood smear showed the presence of many schistocytes and reticulocytosis (Figure 1), and laboratories (Table 1) confirmed hemolysis. Cold agglutinin test, paroxysmal nocturnal hemoglobinuria markers, antinuclear antibody, and direct antibody testing were negative. Fecal pathogen polymerase chain reaction (PCR) testing was positive for EPEC. ADAMTS13 activity level was decreased at 32% (normal is greater than 67%) but did not meet thrombotic thrombocytopenic purpura's (TTP) diagnostic criteria (below 5%‐10%). TTP was of low likelihood as the plasmic score was at two putting patient at low‐risk category. Since suspicion for STEC‐HUS was greater than the suspicion for TTP, it was felt appropriate to defer plasmapheresis therapy until the ADAMTS13 levels resulted. Other etiologies including infection, DIC, HELLP syndrome, severe hypertension, medication interactions, autoimmune conditions, Vit b12 deficiency, dialysis, mechanical causes of shearing, and occult malignancy screening were ruled. Atypical HUS genetic panel was negative, and CH50 and C1 esterase inhibitor levels were within normal limits. All infectious workup with stool/blood/urine cultures and imaging were negative except for EPEC positivity. Urine analysis revealed 3 + urine hemoglobin with no myoglobin and 15RBCs/HPF (ongoing hematuria) and nephrotic‐range proteinuria (2 gm/day) which spoke against rhabdomyolysis and inclined toward an intrinsic kidney process such as HUS.

**TABLE 1:** Laboratory values during hospital course

	Admission	Day before treatment	Day 5	Day 12	Discharge
Labs	Day 1	Day 5	Day 10	Day 22	Day 25
WBC (10*3/µL)	21 430	42 160	9.65	5490	10 360
HGB (g/dL)	14.8	5.0	8.5	8.8	9.7
PLT (10*3/µL)	227	66	340	385	200
Cr (mg/dL)	2.20	3.94	4.45	2.42	2.28
BUN (mg/dL)	37	100	62	26	23
CK (U/L)	10 846	NA	32	NA	NA
LDH (U/L)	NA	3 399	549	515	NA
Reticulocyte Index	0.34	0.09	1.72	30.6	NA
Haptoglobin (mg/dL)	NA	<6	106	163	NA

Note

The patient initially presented with leukocytosis and elevated BUN and Cr After 12 d of treatment, she was stable and hemolysis significantly improved.

Patient was continued on supportive therapy. No indication for use of IV antibiotics was noted. She was started on prednisone 1.5 mg/kg to help with the MAHA component of HUS which improved her anemia and dependence on transfusion. Mentation improved, and repeat blood smear showed significantly fewer schistocytes compared to before. Her CBC and CMP values and hemolysis improved as well (Table 1). She was on hemodialysis for a total of 5 weeks. She can now live independently, but residual symptoms include moderate fatigue, weakness, and decreased cognition.

## 3 DISCUSSION

Documented cases of typical HUS in adult patients are very rare and usually are caused by O157:H7 strains of *E Coli* that can be cultured and/or detected by stool studies that detect the organism's Shiga‐like toxins stx1/stx2.^3^, ^4^ This case is a rare example of HUS in an adult patient whose fecal pathogen PCR was positive for EPEC rather than EHEC.

Enteropathogenic *E Coli* produces watery diarrhea seen in this patient case, whereas EHEC causes bloody diarrhea. EPEC produces an attaching and effacing lesion (A/E) upon the epithelial surface of the small and/or large bowel. It then utilizes a type III secretion system to inject virulence factors into host cells and a type IV bundle‐forming pilus to establish microcolonies. It is hypothesized that EPEC causes watery diarrhea by disrupting the absorptive surfaces of the intestinal microvilli.^5^ EHEC also forms A/E lesions on the intestinal epithelium, as it shares the same chromosomal pathogenicity island as EPEC. However, what distinguishes EHEC is the production of Shiga‐like toxin, which binds to endothelial cells that express Gb3 and enables diffuse spread of the toxin to various cells (including renal glomerular endothelium) that express Gb3. Moreover, EHEC causes bloody diarrhea but EPEC does not because the A subunit of EHEC's Shiga‐like toxin prevents protein synthesis and triggers apoptosis of the host cells.^6^

Typical HUS, atypical HUS, and TTP are all causes of MAHA, but each has distinct mechanisms. tHUS is caused by EHEC's verotoxins or *Shigella's* enterotoxins, which cause vascular damage (specifically to the glomerular endothelium) and thereby increase platelet adhesion and promote microthrombi formation.^7^ In contrast, aHUS is unrelated to *E Coli* or *Shigella* infection and is instead caused by abnormal regulatory genes of the complement pathway. TTP is caused by antibodies disrupting the ADAMTS13 enzyme, resulting in the accumulation of von Willebrand factor multimers that cause MAHA. Another type of HUS that does not fit into the typical or atypical categories is secondary HUS, which may be caused by streptococcal infection or other acquired sources of complement dysregulation such as pregnancy, chemotherapy, malignancy, glomerular disorders, or autoimmune diseases. If the patient has no genetic abnormalities in the complement pathway, no identifiable causes of secondary HUS, and normal ADAMTS13 activity, then the TMA is idiopathic HUS.^8^

Where on the complex spectrum of HUS does this patient's case belong? She did not have an EHEC or *Shigella* infection and no Shiga or Shiga‐like toxins were identified, so her TMA cannot be diagnosed as tHUS. On the other hand, with no genetic abnormality in complement regulation and older age, the patient does not meet the criteria for aHUS either where the median age is in the 20s, although it is notable to know that 50%‐70% of patients will have no known genetic mutation in the complement regulatory proteins.^9^ Since the suspected cause of her TMA is EPEC, her case is best described as HUS secondary to EPEC infection by indirect correlation after extensive rule out of possible differentials inciting a similar picture. Although the possible mechanisms of EPEC‐related HUS have not yet been elucidated, tHUS, aHUS, and TTP are all ultimately consequences of complement system hyperactivation; thus, it is feasible that EPEC’s effect on the host systemic inflammatory and complement pathways could also incite HUS despite lacking EHEC’s Shiga‐like toxins.^10^

## 4 CONCLUSION

Hemolytic‐uremic syndrome has many diverse presentations and etiologies, some of which are still poorly understood. Whereas HUS typically occurs in the pediatric population and is caused by EHEC, this case illustrates how HUS can also be found in adult patients and may be caused by other organisms such as EPEC. A thorough workup is required to identify HUS as the diagnosis and rule out other potential causes of MAHA and thrombocytopenia. Recognizing the different presentations and causes of HUS in adult patients is essential for conducting appropriate diagnostic studies and patient care. We hope that this hypothesis‐generating presentation will encourage the educational society to initiate a pathway that needs further refinement to develop a direct correlation and question what we know so far about this syndrome.

## CONFLICT OF INTEREST

None declared.

## AUTHOR CONTRIBUTIONS

BN: developed the original idea, analyzed the data, prepared the manuscript, and provided additional review. AH and FI: contributed to the development of the paper and prepared the manuscript. BJ: provided critical revision of the manuscript for important intellectual content.

## ETHICAL APPROVAL

Authors declare human ethics approval was not required for this case report.
